# (*S*)-*N*-[1-(5-Benzyl­sulfan­yl-1,3,4-oxa­diazol-2-yl)-2-phenyl­eth­yl]-4-methyl­benzene­sulfonamide

**DOI:** 10.1107/S1600536811040669

**Published:** 2011-10-08

**Authors:** Tayyaba Syed, Shahid Hameed, Peter G. Jones

**Affiliations:** a Department of Chemistry, Quaid-i-Azam University, Islamabad 45320, Pakistan; b Institut for Anorganische und Analytische Chemie, Technische Universität Braunschweig, Hagenring 30, 38106 Braunschweig, Germany

## Abstract

The title compound, C_24_H_23_N_3_O_3_S_2_, crystallizes with two independent mol­ecules in the asymmetric unit. They differ essentially in the orientation of the tolyl rings, between which there is π–π stacking (centroid–centroid distance = 3.01 Å). The absolute configuration was confirmed by the determination of the Flack parameter [*x* = 0.008 (9)]. In the crystal, mol­ecules are connected by two classical N—H⋯N hydrogen bonds and two weak but very short C—H⋯O_sulfon­yl_ inter­actions, forming layers lying parallel to the *bc* plane.

## Related literature

For the biological activity of substituted-1,3,4-oxadiazo­les, see: Aboraia *et al.* (2006 ▶); Akhtar *et al.* (2008 ▶, 2010 ▶); Iqbal *et al.* (2006 ▶); Syed *et al.* (2011*a* ▶); Zahid *et al.* (2009 ▶); Zareef *et al.* (2007 ▶); Zarghi *et al.* (2005 ▶). For the crystal structure of the 4-methyl derivative (which has a methyl instead of a phenylmethyl substituent at C6), see: Syed *et al.* (2011*b* ▶). For the synthesis of the title compound, see: Syed *et al.* (2011*a* ▶). For information concerning the program *RPLUTO*, see: CCDC (2007 ▶).
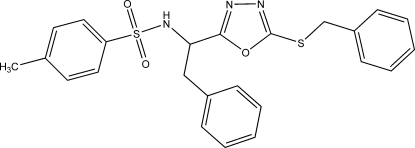


## Experimental

### 

#### Crystal data

C_24_H_23_N_3_O_3_S_2_
*M* *_r_* = 465.57
Monoclinic, 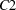

*a* = 41.128 (2) Å
*b* = 5.7205 (5) Å
*c* = 18.9783 (11) Å
β = 90.940 (4)°
*V* = 4464.5 (5) Å^3^
*Z* = 8
Cu *K*α radiation
μ = 2.43 mm^−1^
*T* = 100 K
0.25 × 0.08 × 0.03 mm

#### Data collection

Oxford Diffraction Xcalibur Nova A diffractometer
Absorption correction: multi-scan (*CrysAlis PRO*; Oxford Diffraction, 2010 ▶) *T*_min_ = 0.740, *T*_max_ = 1.000
42976 measured reflections
8447 independent reflections
7718 reflections with *I* > 2σ(*I*)
*R*_int_ = 0.049

#### Refinement

*R*[*F* ^2^ > 2σ(*F* ^2^)] = 0.031
*wR*(*F* ^2^) = 0.080
*S* = 1.04
8447 reflections
587 parameters
1 restraint
H atoms treated by a mixture of independent and constrained refinement
Δρ_max_ = 0.27 e Å^−3^
Δρ_min_ = −0.21 e Å^−3^
Absolute structure: Flack (1983 ▶), 3306 Friedel pairs
Flack parameter: 0.008 (9)

### 

Data collection: *CrysAlis PRO* (Oxford Diffraction, 2010 ▶); cell refinement: *CrysAlis PRO*; data reduction: *CrysAlis PRO*; program(s) used to solve structure: *SHELXS97* (Sheldrick, 2008 ▶); program(s) used to refine structure: *SHELXL97* (Sheldrick, 2008 ▶); molecular graphics: *XP* (Siemens, 1994 ▶); software used to prepare material for publication: *SHELXL97*.

## Supplementary Material

Crystal structure: contains datablock(s) I, global. DOI: 10.1107/S1600536811040669/su2319sup1.cif

Structure factors: contains datablock(s) I. DOI: 10.1107/S1600536811040669/su2319Isup2.hkl

Supplementary material file. DOI: 10.1107/S1600536811040669/su2319Isup3.cml

Additional supplementary materials:  crystallographic information; 3D view; checkCIF report

## Figures and Tables

**Table 1:** Hydrogen-bond geometry (Å, °)

*D*—H⋯*A*	*D*—H	H⋯*A*	*D*⋯*A*	*D*—H⋯*A*
N5—H05⋯N4^i^	0.79 (3)	2.22 (3)	3.003 (2)	178 (3)
N5′—H05′⋯N4′^i^	0.82 (3)	2.23 (3)	3.048 (2)	171 (2)
C21—H21*B*⋯O2′^ii^	0.99	2.32	3.249 (2)	155
C21′—H21*D*⋯O2^iii^	0.99	2.23	3.145 (2)	154
C19—H19⋯O3^iv^	0.95	2.50	3.352 (2)	150

Symmetry codes: (i) 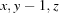; (ii) 
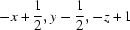
; (iii) 
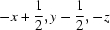
; (iv) 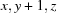.

**Fig. 1.**
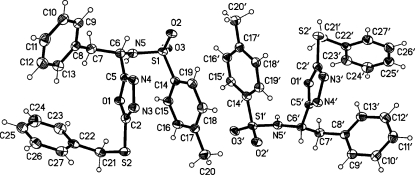
The molecular structure of the title compound, showing the numbering scheme for the two independent molecules (1 left; 2 right). Displacement ellipsoids are drawn at the 50% probability levels.

**Fig. 2.**
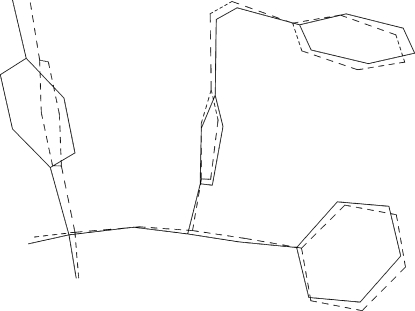
A least-squares fit of the two independent molecules (molecule 1 dotted lines). All non-H atoms were fitted except the ring atoms (C15–C20) on the left-hand side of the figure.

**Fig. 3.**
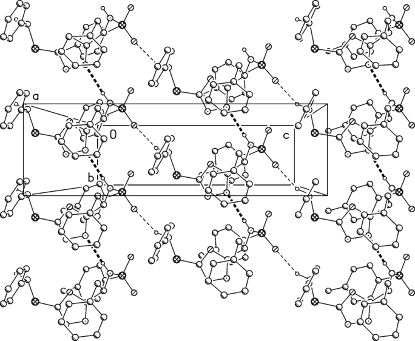
Crystal packing of the title compound, viewed perpendicular to the bc plane in the region x≈ 7/8. Thick dashed lines represent classical and thin dashed lines "weak" hydrogen bonds. Hydrogen atoms not involved in these interactions have been omitted for clarity. The three vertical rows of molecules are composed of molecules 2, 1, 2, respectively.

## supplementary crystallographic information

### Comment

The literature contains many reports of a variety of biological activities of substituted-1,3,4-oxadiazoles. These include anti-convulsant (Zarghi *et al.*, 2005), anti-proliferative (Zahid *et al.*, 2009), anti-tumour and anti-viral (Akhtar *et al.*, 2008), anti-bacterial (Iqbal *et al.*, 2006), urease inhibition (Akhtar *et al.*, 2010), and anti-mitotic (Aboraia *et al.*, 2006) activities. Some sulfonamide-bearing 1,3,4-oxadiazoles have also been reported in the literature to show anti-HIV (Syed *et al.*, 2011*a*), and anti-microbial (Zareef *et al.*, 2007) activities. The title compound was synthesized in our laboratory to explore its anti-HIV and anti-HCV activities and herein we report on its crystal structure.

The molecular structure of the two independent molecules (1 and 2) of the enantiomerically pure title compound are shown in Fig. 1. The two molecules are related by a pseudo twofold axis. The main difference between the two molecules is the rotation of the tolyl group; the corresponding torsion angles are N5—S1—C14—C15 61.4 (2), and N5'—S1'—C14'—C15' 100.2 (2)°. A least-squares fit of both molecules, ignoring atoms (C15–C20), gave an r.m.s. deviation of 0.19 Å (Fig. 2). The large number of single bonds means that the molecules have a considerable number of torsional degrees of freedom. The conformation actually adopted is a flattened form in which all rings are arranged to correspond approximately to the smallest dimension of the molecular "box". This was calculated, [RPLUTO; CCDC, 2007], to be 7.9Å for molecule 1 and 7.7 Å for molecule 2.

Within the asymmetric unit, significant contacts are the intramolecular C13-H13···*Cg*(C22–27) 2.91 Å, and the π···π stacking between rings (C14–C19) and (C14'–C19') with a centroid-centroid distance of 3.01 Å . The former necessitates a suitable orientation of the ring (C22–C27), associated with the torsion angle N3—C2—S2—C21 155.8 (2), and N3'—C2'—S2'—C2' 1154.3 (2)°. In the corresponding compound with a methyl instead of a phenylmethyl substituent at C6 (Syed *et al.*, 2011*b*), this ring is rotated in the opposite direction, towards the tolyl group, with torsion angle N—C—S—C being -3.6 (2)°. There the π···π contact has an interplanar angle of 8.2 (1)° and a *Cg*···*Cg* distance of 3.75 Å; the ring offset was estimated to be *ca* 1.5 Å.

In the crystal of the title compound, the molecular packing is largely determined by two short classical N-H···N hydrogen bonds, together with two "weak" but very short C-H···O_sulfonyl_ interactions (see Table 1 for details). The combinaton of these interactions leads to the formation of layers lying parallel to the *bc* plane (Fig. 3).

### Experimental

The synthesis and spectral data of the compound under study have been described previously by our laboratory (Syed *et al.*, 2011*a*).

### Refinement

The H atoms at N5 and N5' were located in a difference Fourier map and were refined freely. The C-bound H atoms were introduced at calculated positions and refined using a riding model: C-H = 0.95, 0.98, 0.99, and 1.00 Å, for aromatic, methyl, methylene and methine H atoms, respectively, with *U*_iso_(H) = k × *U*_eq_(C), where k = 1.5 for methyl H atoms and k = 1.2 for all other H atoms. The absolute configuration (*S* at C6 and C6') was established by resonant scattering; the Flack parameter is 0.008 (9) and the Hooft parameter is 0.013 (4).

### Crystal data

**Table:**

C_24_H_23_N_3_O_3_S_2_	*F*(000) = 1952
*M**_r_* = 465.57	*D*_x_ = 1.385 Mg m^−^^3^
Monoclinic, *C*2	Cu *K*α radiation, λ = 1.54184 Å
*a* = 41.128 (2) Å	Cell parameters from 19219 reflections
*b* = 5.7205 (5) Å	θ = 3.1–75.7°
*c* = 18.9783 (11) Å	µ = 2.43 mm^−^^1^
β = 90.940 (4)°	*T* = 100 K
*V* = 4464.5 (5) Å^3^	Lath, colourless
*Z* = 8	0.25 × 0.08 × 0.03 mm

### Data collection

**Table:**

Oxford Diffraction Xcalibur Nova A diffractometer	8447 independent reflections
Radiation source: Nova (Cu) X-ray Source	7718 reflections with *I* > 2σ(*I*)
mirror	*R*_int_ = 0.049
Detector resolution: 10.3543 pixels mm^-1^	θ_max_ = 76.0°, θ_min_ = 3.1°
ω–scan	*h* = −50→51
Absorption correction: multi-scan (*CrysAlis PRO*; Oxford Diffraction, 2010)	*k* = −7→6
*T*_min_ = 0.740, *T*_max_ = 1.000	*l* = −23→23
42976 measured reflections	

### Refinement

**Table:**

Refinement on *F*^2^	Secondary atom site location: difference Fourier map
Least-squares matrix: full	Hydrogen site location: inferred from neighbouring sites
*R*[*F*^2^ > 2σ(*F*^2^)] = 0.031	H atoms treated by a mixture of independent and constrained refinement
*wR*(*F*^2^) = 0.080	*w* = 1/[σ^2^(*F*_o_^2^) + (0.0523*P*)^2^] where *P* = (*F*_o_^2^ + 2*F*_c_^2^)/3
*S* = 1.04	(Δ/σ)_max_ = 0.001
8447 reflections	Δρ_max_ = 0.27 e Å^−^^3^
587 parameters	Δρ_min_ = −0.21 e Å^−^^3^
1 restraint	Absolute structure: Flack (1983), 3306 Friedel pairs
Primary atom site location: structure-invariant direct methods	Flack parameter: 0.008 (9)

### Special details

**Table:**

Geometry. All e.s.d.'s (except the e.s.d. in the dihedral angle between two l.s. planes) are estimated using the full covariance matrix. The cell e.s.d.'s are taken into account individually in the estimation of e.s.d.'s in distances, angles and torsion angles; correlations between e.s.d.'s in cell parameters are only used when they are defined by crystal symmetry. An approximate (isotropic) treatment of cell e.s.d.'s is used for estimating e.s.d.'s involving l.s. planes.
Refinement. Refinement of *F*^2^ against ALL reflections. The weighted *R*-factor *wR* and goodness of fit *S* are based on *F*^2^, conventional *R*-factors *R* are based on *F*, with *F* set to zero for negative *F*^2^. The threshold expression of *F*^2^ > σ(*F*^2^) is used only for calculating *R*-factors(gt) *etc*. and is not relevant to the choice of reflections for refinement. *R*-factors based on *F*^2^ are statistically about twice as large as those based on *F*, and *R*- factors based on ALL data will be even larger.

### Fractional atomic coordinates and isotropic or equivalent isotropic displacement parameters (Å^2^)

**Table:**

	*x*	*y*	*z*	*U*_iso_*/*U*_eq_	
S1	0.166517 (10)	0.47595 (8)	0.20778 (2)	0.02157 (10)	
S2	0.167054 (11)	0.78799 (10)	0.51291 (2)	0.02831 (11)	
O1	0.13716 (3)	0.6557 (2)	0.39040 (6)	0.0230 (3)	
C2	0.14887 (4)	0.8364 (3)	0.43097 (9)	0.0230 (4)	
N3	0.14640 (4)	1.0359 (3)	0.40067 (8)	0.0266 (3)	
N4	0.13278 (4)	0.9900 (3)	0.33309 (8)	0.0229 (3)	
C5	0.12768 (4)	0.7679 (4)	0.32999 (9)	0.0209 (4)	
N5	0.13242 (4)	0.4323 (3)	0.24788 (8)	0.0232 (3)	
H05	0.1322 (6)	0.318 (5)	0.2709 (12)	0.030 (6)*	
O2	0.16115 (3)	0.6639 (3)	0.15920 (6)	0.0261 (3)	
O3	0.17624 (3)	0.2503 (3)	0.18271 (6)	0.0268 (3)	
C6	0.11270 (4)	0.6305 (3)	0.27056 (10)	0.0227 (4)	
H6	0.1097	0.7381	0.2294	0.027*	
C7	0.07905 (5)	0.5358 (4)	0.29039 (10)	0.0272 (4)	
H7A	0.0699	0.4442	0.2505	0.033*	
H7B	0.0815	0.4296	0.3313	0.033*	
C8	0.05586 (4)	0.7294 (4)	0.30833 (10)	0.0255 (4)	
C9	0.04399 (4)	0.8782 (4)	0.25553 (10)	0.0279 (4)	
H9	0.0506	0.8553	0.2083	0.033*	
C10	0.02279 (5)	1.0582 (4)	0.27069 (11)	0.0308 (4)	
H10	0.0147	1.1566	0.2341	0.037*	
C11	0.01329 (5)	1.0945 (4)	0.34032 (12)	0.0345 (5)	
H11	−0.0011	1.2188	0.3514	0.041*	
C12	0.02501 (5)	0.9474 (5)	0.39293 (11)	0.0387 (5)	
H12	0.0184	0.9699	0.4401	0.046*	
C13	0.04630 (5)	0.7682 (5)	0.37735 (11)	0.0348 (5)	
H13	0.0545	0.6708	0.4141	0.042*	
C14	0.19511 (4)	0.5712 (4)	0.27153 (9)	0.0227 (4)	
C15	0.20295 (5)	0.4229 (4)	0.32732 (10)	0.0269 (4)	
H15	0.1929	0.2739	0.3309	0.032*	
C16	0.22558 (5)	0.4952 (4)	0.37751 (10)	0.0305 (4)	
H16	0.2311	0.3940	0.4155	0.037*	
C17	0.24035 (4)	0.7134 (4)	0.37338 (10)	0.0278 (4)	
C18	0.23229 (5)	0.8570 (4)	0.31670 (10)	0.0278 (4)	
H18	0.2426	1.0049	0.3127	0.033*	
C19	0.20954 (4)	0.7895 (4)	0.26571 (9)	0.0258 (4)	
H19	0.2040	0.8907	0.2277	0.031*	
C20	0.26377 (5)	0.7947 (5)	0.42984 (10)	0.0376 (5)	
H20A	0.2517	0.8419	0.4716	0.056*	
H20B	0.2762	0.9281	0.4124	0.056*	
H20C	0.2787	0.6670	0.4423	0.056*	
C21	0.14683 (5)	0.5189 (4)	0.53937 (10)	0.0292 (4)	
H21A	0.1511	0.3969	0.5037	0.035*	
H21B	0.1565	0.4655	0.5846	0.035*	
C22	0.11060 (5)	0.5414 (4)	0.54782 (9)	0.0275 (4)	
C23	0.09059 (6)	0.3627 (4)	0.52384 (11)	0.0358 (5)	
H23	0.0998	0.2311	0.5011	0.043*	
C24	0.05716 (6)	0.3738 (5)	0.53275 (13)	0.0428 (6)	
H24	0.0438	0.2485	0.5169	0.051*	
C25	0.04327 (5)	0.5660 (4)	0.56454 (11)	0.0367 (5)	
H25	0.0204	0.5744	0.5701	0.044*	
C26	0.06304 (5)	0.7455 (4)	0.58817 (10)	0.0347 (5)	
H26	0.0537	0.8783	0.6100	0.042*	
C27	0.09657 (5)	0.7340 (4)	0.58035 (10)	0.0318 (5)	
H27	0.1099	0.8580	0.5973	0.038*	
S1'	0.321235 (10)	0.48913 (9)	0.28913 (2)	0.02305 (10)	
S2'	0.339409 (11)	0.78665 (9)	−0.01838 (2)	0.02753 (11)	
O1'	0.36121 (3)	0.6560 (2)	0.11112 (6)	0.0220 (3)	
C2'	0.35354 (4)	0.8379 (4)	0.06659 (10)	0.0237 (4)	
N3'	0.35610 (4)	1.0389 (3)	0.09662 (8)	0.0252 (3)	
N4'	0.36541 (4)	0.9927 (3)	0.16741 (8)	0.0233 (3)	
C5'	0.36816 (4)	0.7694 (4)	0.17275 (9)	0.0222 (4)	
N5'	0.35627 (4)	0.4383 (3)	0.25289 (8)	0.0233 (3)	
H05'	0.3569 (5)	0.323 (5)	0.2274 (11)	0.023 (6)*	
O2'	0.32614 (3)	0.6908 (3)	0.33283 (7)	0.0294 (3)	
O3'	0.31089 (3)	0.2714 (3)	0.31844 (7)	0.0296 (3)	
C6'	0.37862 (4)	0.6282 (3)	0.23546 (9)	0.0224 (4)	
H6'	0.3804	0.7352	0.2770	0.027*	
C7'	0.41243 (4)	0.5204 (4)	0.22288 (10)	0.0258 (4)	
H7'1	0.4202	0.4406	0.2663	0.031*	
H7'2	0.4108	0.4025	0.1848	0.031*	
C8'	0.43648 (4)	0.7080 (4)	0.20266 (10)	0.0250 (4)	
C9'	0.44705 (4)	0.8726 (4)	0.25207 (10)	0.0273 (4)	
H9'	0.4398	0.8620	0.2992	0.033*	
C10'	0.46791 (5)	1.0508 (4)	0.23364 (11)	0.0306 (4)	
H10'	0.4750	1.1612	0.2680	0.037*	
C11'	0.47863 (5)	1.0687 (4)	0.16422 (12)	0.0344 (5)	
H11'	0.4929	1.1910	0.1511	0.041*	
C12'	0.46813 (5)	0.9063 (4)	0.11491 (12)	0.0359 (5)	
H12'	0.4753	0.9178	0.0677	0.043*	
C13'	0.44723 (5)	0.7261 (4)	0.13372 (10)	0.0314 (5)	
H13'	0.4403	0.6152	0.0994	0.038*	
C14'	0.29307 (5)	0.5646 (4)	0.22125 (10)	0.0239 (4)	
C15'	0.27134 (4)	0.3988 (4)	0.19568 (10)	0.0251 (4)	
H15'	0.2708	0.2471	0.2160	0.030*	
C16'	0.25036 (4)	0.4563 (4)	0.13994 (10)	0.0270 (4)	
H16'	0.2352	0.3435	0.1231	0.032*	
C17'	0.25124 (4)	0.6745 (4)	0.10860 (10)	0.0257 (4)	
C18'	0.27325 (4)	0.8394 (4)	0.13545 (10)	0.0255 (4)	
H18'	0.2744	0.9894	0.1142	0.031*	
C19'	0.29351 (4)	0.7883 (4)	0.19256 (9)	0.0254 (4)	
H19'	0.3075	0.9047	0.2118	0.030*	
C20'	0.22829 (5)	0.7398 (4)	0.04908 (10)	0.0320 (5)	
H20D	0.2161	0.6012	0.0338	0.048*	
H20E	0.2407	0.8009	0.0095	0.048*	
H20F	0.2131	0.8598	0.0652	0.048*	
C21'	0.35966 (5)	0.5122 (4)	−0.03843 (10)	0.0277 (4)	
H21C	0.3552	0.3993	−0.0003	0.033*	
H21D	0.3501	0.4483	−0.0826	0.033*	
C22'	0.39605 (5)	0.5307 (4)	−0.04671 (9)	0.0261 (4)	
C23'	0.41558 (5)	0.3493 (4)	−0.02205 (11)	0.0331 (5)	
H23'	0.4061	0.2206	0.0015	0.040*	
C24'	0.44899 (6)	0.3552 (5)	−0.03170 (12)	0.0386 (5)	
H24'	0.4621	0.2291	−0.0154	0.046*	
C25'	0.46332 (5)	0.5443 (4)	−0.06506 (11)	0.0339 (5)	
H25'	0.4862	0.5491	−0.0711	0.041*	
C26'	0.44391 (5)	0.7253 (4)	−0.08939 (11)	0.0347 (5)	
H26'	0.4535	0.8551	−0.1123	0.042*	
C27'	0.41052 (5)	0.7189 (4)	−0.08060 (10)	0.0318 (4)	
H27'	0.3974	0.8438	−0.0978	0.038*	

### Atomic displacement parameters (Å^2^)

**Table:**

	*U*^11^	*U*^22^	*U*^33^	*U*^12^	*U*^13^	*U*^23^
S1	0.0286 (2)	0.0198 (2)	0.01621 (18)	0.00177 (19)	−0.00120 (14)	0.00067 (18)
S2	0.0317 (2)	0.0313 (3)	0.0218 (2)	−0.0015 (2)	−0.00495 (16)	−0.0002 (2)
O1	0.0299 (6)	0.0196 (7)	0.0196 (6)	−0.0007 (5)	−0.0029 (5)	0.0012 (5)
C2	0.0249 (8)	0.0210 (11)	0.0232 (9)	−0.0003 (7)	0.0023 (7)	−0.0021 (7)
N3	0.0318 (8)	0.0233 (9)	0.0245 (7)	−0.0026 (6)	−0.0021 (6)	−0.0026 (7)
N4	0.0289 (7)	0.0197 (8)	0.0202 (7)	−0.0016 (7)	−0.0003 (5)	0.0016 (7)
C5	0.0222 (8)	0.0212 (10)	0.0192 (8)	0.0021 (7)	−0.0005 (6)	0.0029 (7)
N5	0.0305 (8)	0.0164 (9)	0.0225 (8)	0.0001 (6)	−0.0030 (6)	0.0010 (7)
O2	0.0349 (7)	0.0243 (8)	0.0192 (6)	0.0023 (6)	−0.0033 (5)	0.0043 (6)
O3	0.0355 (7)	0.0242 (8)	0.0207 (6)	0.0034 (6)	−0.0013 (5)	−0.0037 (5)
C6	0.0256 (9)	0.0189 (10)	0.0237 (8)	0.0018 (7)	−0.0015 (7)	0.0023 (7)
C7	0.0282 (9)	0.0237 (11)	0.0296 (9)	−0.0020 (8)	−0.0031 (7)	0.0026 (8)
C8	0.0225 (8)	0.0280 (11)	0.0260 (9)	−0.0042 (8)	−0.0019 (7)	−0.0001 (8)
C9	0.0243 (9)	0.0322 (11)	0.0272 (9)	−0.0019 (8)	−0.0014 (7)	0.0020 (8)
C10	0.0265 (9)	0.0317 (12)	0.0339 (10)	0.0001 (8)	−0.0031 (8)	0.0026 (9)
C11	0.0277 (9)	0.0323 (13)	0.0436 (12)	0.0013 (9)	0.0024 (8)	−0.0072 (10)
C12	0.0370 (11)	0.0508 (16)	0.0285 (10)	−0.0019 (10)	0.0046 (8)	−0.0098 (10)
C13	0.0337 (10)	0.0422 (14)	0.0284 (9)	0.0001 (10)	−0.0006 (8)	0.0033 (10)
C14	0.0261 (8)	0.0227 (10)	0.0192 (8)	0.0023 (7)	0.0009 (7)	−0.0016 (7)
C15	0.0301 (9)	0.0273 (12)	0.0232 (9)	−0.0019 (7)	−0.0021 (7)	0.0028 (8)
C16	0.0338 (10)	0.0367 (12)	0.0208 (8)	0.0016 (10)	−0.0042 (7)	0.0036 (9)
C17	0.0243 (9)	0.0367 (12)	0.0224 (9)	0.0013 (8)	0.0013 (7)	−0.0077 (8)
C18	0.0295 (9)	0.0241 (11)	0.0300 (10)	−0.0023 (8)	0.0023 (7)	−0.0045 (8)
C19	0.0293 (9)	0.0232 (10)	0.0249 (8)	0.0018 (8)	0.0019 (7)	0.0019 (8)
C20	0.0317 (10)	0.0518 (15)	0.0291 (10)	−0.0014 (11)	−0.0030 (8)	−0.0094 (11)
C21	0.0369 (10)	0.0283 (12)	0.0224 (9)	0.0026 (8)	−0.0010 (7)	0.0053 (8)
C22	0.0391 (10)	0.0246 (11)	0.0188 (8)	0.0017 (8)	−0.0013 (7)	0.0073 (8)
C23	0.0467 (12)	0.0264 (12)	0.0344 (11)	−0.0006 (10)	0.0009 (9)	−0.0004 (9)
C24	0.0468 (13)	0.0366 (14)	0.0448 (13)	−0.0116 (11)	−0.0028 (10)	0.0031 (11)
C25	0.0347 (11)	0.0443 (14)	0.0310 (10)	−0.0019 (10)	−0.0005 (8)	0.0127 (10)
C26	0.0392 (10)	0.0382 (14)	0.0267 (9)	0.0048 (10)	0.0034 (8)	0.0026 (9)
C27	0.0368 (10)	0.0327 (13)	0.0259 (9)	−0.0011 (9)	−0.0034 (7)	−0.0005 (9)
S1'	0.0299 (2)	0.0215 (2)	0.01781 (19)	−0.0008 (2)	−0.00022 (15)	−0.00065 (18)
S2'	0.0338 (2)	0.0290 (3)	0.0197 (2)	0.0039 (2)	−0.00464 (16)	−0.0004 (2)
O1'	0.0285 (6)	0.0186 (7)	0.0188 (6)	0.0009 (5)	−0.0022 (5)	−0.0009 (5)
C2'	0.0261 (9)	0.0229 (11)	0.0221 (8)	0.0032 (7)	0.0000 (7)	0.0040 (7)
N3'	0.0317 (8)	0.0211 (9)	0.0228 (7)	0.0003 (6)	−0.0011 (6)	0.0014 (6)
N4'	0.0305 (7)	0.0193 (8)	0.0200 (7)	0.0005 (7)	−0.0005 (5)	−0.0006 (7)
C5'	0.0247 (8)	0.0228 (10)	0.0189 (8)	−0.0006 (8)	−0.0016 (6)	−0.0032 (8)
N5'	0.0305 (8)	0.0173 (9)	0.0222 (7)	−0.0011 (6)	−0.0017 (6)	−0.0021 (7)
O2'	0.0363 (7)	0.0295 (8)	0.0224 (6)	0.0004 (6)	−0.0004 (5)	−0.0064 (6)
O3'	0.0368 (7)	0.0288 (8)	0.0232 (6)	−0.0039 (6)	0.0012 (5)	0.0065 (6)
C6'	0.0269 (9)	0.0188 (10)	0.0215 (8)	−0.0015 (7)	−0.0039 (7)	−0.0010 (7)
C7'	0.0271 (9)	0.0225 (11)	0.0276 (9)	0.0030 (8)	−0.0034 (7)	−0.0007 (8)
C8'	0.0234 (8)	0.0252 (11)	0.0265 (9)	0.0037 (7)	−0.0031 (7)	0.0000 (8)
C9'	0.0252 (9)	0.0302 (11)	0.0266 (9)	0.0045 (8)	−0.0004 (7)	−0.0024 (8)
C10'	0.0264 (9)	0.0299 (12)	0.0356 (10)	0.0004 (8)	−0.0014 (8)	−0.0058 (9)
C11'	0.0304 (10)	0.0294 (12)	0.0435 (12)	−0.0003 (9)	0.0030 (9)	0.0059 (10)
C12'	0.0399 (11)	0.0377 (14)	0.0303 (10)	0.0034 (9)	0.0023 (8)	0.0055 (9)
C13'	0.0312 (9)	0.0364 (13)	0.0266 (9)	0.0038 (9)	−0.0018 (7)	−0.0049 (9)
C14'	0.0279 (9)	0.0212 (10)	0.0226 (8)	0.0019 (7)	0.0020 (7)	−0.0015 (7)
C15'	0.0293 (9)	0.0218 (10)	0.0244 (9)	0.0007 (7)	0.0035 (7)	0.0002 (8)
C16'	0.0267 (9)	0.0269 (11)	0.0272 (9)	−0.0014 (8)	−0.0008 (7)	−0.0026 (8)
C17'	0.0234 (9)	0.0278 (11)	0.0261 (9)	0.0023 (8)	0.0032 (7)	−0.0023 (8)
C18'	0.0278 (9)	0.0221 (11)	0.0268 (9)	0.0004 (7)	0.0030 (7)	0.0013 (7)
C19'	0.0264 (8)	0.0232 (10)	0.0265 (8)	−0.0002 (8)	0.0008 (7)	−0.0018 (8)
C20'	0.0318 (10)	0.0330 (13)	0.0311 (10)	−0.0006 (9)	−0.0048 (8)	0.0000 (9)
C21'	0.0350 (10)	0.0265 (11)	0.0216 (8)	−0.0005 (8)	−0.0028 (7)	−0.0067 (8)
C22'	0.0365 (10)	0.0248 (11)	0.0171 (8)	−0.0010 (8)	−0.0001 (7)	−0.0056 (8)
C23'	0.0421 (11)	0.0256 (12)	0.0315 (10)	0.0039 (9)	0.0011 (8)	0.0005 (9)
C24'	0.0401 (12)	0.0360 (13)	0.0397 (12)	0.0119 (10)	−0.0015 (9)	−0.0002 (10)
C25'	0.0340 (10)	0.0389 (13)	0.0288 (10)	0.0007 (9)	0.0008 (8)	−0.0074 (9)
C26'	0.0391 (11)	0.0352 (13)	0.0299 (10)	−0.0046 (9)	0.0025 (8)	−0.0009 (9)
C27'	0.0381 (10)	0.0284 (12)	0.0288 (10)	0.0006 (9)	−0.0015 (8)	0.0005 (8)

### Geometric parameters (Å, °)

**Table:**

S1—O2	1.4312 (14)	C15'—C16'	1.394 (3)
S1—O3	1.4351 (15)	C16'—C17'	1.383 (3)
S1—N5	1.6256 (17)	C17'—C18'	1.398 (3)
S1—C14	1.7597 (19)	C17'—C20'	1.507 (3)
S2—C2	1.7368 (19)	C18'—C19'	1.387 (3)
S2—C21	1.824 (2)	C21'—C22'	1.511 (3)
O1—C5	1.365 (2)	C22'—C23'	1.389 (3)
O1—C2	1.371 (2)	C22'—C27'	1.392 (3)
C2—N3	1.281 (3)	C23'—C24'	1.389 (3)
N3—N4	1.416 (2)	C24'—C25'	1.390 (3)
N4—C5	1.289 (3)	C25'—C26'	1.382 (3)
C5—C6	1.499 (3)	C26'—C27'	1.387 (3)
N5—C6	1.463 (2)	N5—H05	0.79 (3)
C6—C7	1.539 (3)	C6—H6	1.0000
C7—C8	1.504 (3)	C7—H7A	0.9900
C8—C13	1.392 (3)	C7—H7B	0.9900
C8—C9	1.397 (3)	C9—H9	0.9500
C9—C10	1.383 (3)	C10—H10	0.9500
C10—C11	1.400 (3)	C11—H11	0.9500
C11—C12	1.386 (3)	C12—H12	0.9500
C12—C13	1.383 (3)	C13—H13	0.9500
C14—C19	1.388 (3)	C15—H15	0.9500
C14—C15	1.391 (3)	C16—H16	0.9500
C15—C16	1.384 (3)	C18—H18	0.9500
C16—C17	1.391 (3)	C19—H19	0.9500
C17—C18	1.389 (3)	C20—H20A	0.9800
C17—C20	1.503 (3)	C20—H20B	0.9800
C18—C19	1.390 (3)	C20—H20C	0.9800
C21—C22	1.507 (3)	C21—H21A	0.9900
C22—C23	1.385 (3)	C21—H21B	0.9900
C22—C27	1.393 (3)	C23—H23	0.9500
C23—C24	1.389 (3)	C24—H24	0.9500
C24—C25	1.382 (4)	C25—H25	0.9500
C25—C26	1.380 (3)	C26—H26	0.9500
C26—C27	1.391 (3)	C27—H27	0.9500
S1'—O3'	1.4316 (15)	N5'—H05'	0.82 (3)
S1'—O2'	1.4331 (15)	C6'—H6'	1.0000
S1'—N5'	1.6329 (16)	C7'—H7'1	0.9900
S1'—C14'	1.772 (2)	C7'—H7'2	0.9900
S2'—C2'	1.7302 (19)	C9'—H9'	0.9500
S2'—C21'	1.820 (2)	C10'—H10'	0.9500
O1'—C5'	1.364 (2)	C11'—H11'	0.9500
O1'—C2'	1.374 (2)	C12'—H12'	0.9500
C2'—N3'	1.287 (3)	C13'—H13'	0.9500
N3'—N4'	1.416 (2)	C15'—H15'	0.9500
N4'—C5'	1.286 (3)	C16'—H16'	0.9500
C5'—C6'	1.496 (3)	C18'—H18'	0.9500
N5'—C6'	1.465 (2)	C19'—H19'	0.9500
C6'—C7'	1.543 (3)	C20'—H20D	0.9800
C7'—C8'	1.513 (3)	C20'—H20E	0.9800
C8'—C13'	1.392 (3)	C20'—H20F	0.9800
C8'—C9'	1.393 (3)	C21'—H21C	0.9900
C9'—C10'	1.381 (3)	C21'—H21D	0.9900
C10'—C11'	1.400 (3)	C23'—H23'	0.9500
C11'—C12'	1.383 (3)	C24'—H24'	0.9500
C12'—C13'	1.392 (3)	C25'—H25'	0.9500
C14'—C15'	1.385 (3)	C26'—H26'	0.9500
C14'—C19'	1.391 (3)	C27'—H27'	0.9500
			
O2—S1—O3	120.22 (8)	S1—N5—H05	113.8 (17)
O2—S1—N5	106.96 (8)	N5—C6—H6	108.0
O3—S1—N5	105.33 (8)	C5—C6—H6	108.0
O2—S1—C14	107.74 (9)	C7—C6—H6	108.0
O3—S1—C14	108.64 (9)	C8—C7—H7A	109.2
N5—S1—C14	107.31 (8)	C6—C7—H7A	109.2
C2—S2—C21	100.92 (9)	C8—C7—H7B	109.2
C5—O1—C2	102.11 (15)	C6—C7—H7B	109.2
N3—C2—O1	113.26 (16)	H7A—C7—H7B	107.9
N3—C2—S2	125.00 (15)	C10—C9—H9	119.4
O1—C2—S2	121.64 (14)	C8—C9—H9	119.4
C2—N3—N4	105.59 (16)	C9—C10—H10	120.2
C5—N4—N3	106.60 (15)	C11—C10—H10	120.2
N4—C5—O1	112.41 (16)	C12—C11—H11	120.3
N4—C5—C6	128.00 (17)	C10—C11—H11	120.3
O1—C5—C6	119.57 (17)	C13—C12—H12	119.7
C6—N5—S1	120.36 (13)	C11—C12—H12	119.7
N5—C6—C5	113.87 (15)	C12—C13—H13	119.7
N5—C6—C7	107.71 (16)	C8—C13—H13	119.7
C5—C6—C7	111.15 (15)	C16—C15—H15	120.4
C8—C7—C6	111.85 (16)	C14—C15—H15	120.4
C13—C8—C9	118.5 (2)	C15—C16—H16	119.4
C13—C8—C7	121.37 (19)	C17—C16—H16	119.4
C9—C8—C7	120.12 (17)	C17—C18—H18	119.2
C10—C9—C8	121.25 (18)	C19—C18—H18	119.2
C9—C10—C11	119.6 (2)	C14—C19—H19	120.7
C12—C11—C10	119.4 (2)	C18—C19—H19	120.7
C13—C12—C11	120.64 (19)	C17—C20—H20A	109.5
C12—C13—C8	120.6 (2)	C17—C20—H20B	109.5
C19—C14—C15	121.02 (18)	H20A—C20—H20B	109.5
C19—C14—S1	120.38 (15)	C17—C20—H20C	109.5
C15—C14—S1	118.60 (16)	H20A—C20—H20C	109.5
C16—C15—C14	119.1 (2)	H20B—C20—H20C	109.5
C15—C16—C17	121.26 (19)	C22—C21—H21A	108.7
C18—C17—C16	118.38 (18)	S2—C21—H21A	108.7
C18—C17—C20	120.8 (2)	C22—C21—H21B	108.7
C16—C17—C20	120.8 (2)	S2—C21—H21B	108.7
C17—C18—C19	121.6 (2)	H21A—C21—H21B	107.6
C14—C19—C18	118.59 (18)	C22—C23—H23	119.7
C22—C21—S2	114.43 (15)	C24—C23—H23	119.7
C23—C22—C27	118.79 (19)	C25—C24—H24	119.8
C23—C22—C21	119.0 (2)	C23—C24—H24	119.8
C27—C22—C21	122.16 (19)	C26—C25—H25	120.4
C22—C23—C24	120.7 (2)	C24—C25—H25	120.4
C25—C24—C23	120.4 (2)	C25—C26—H26	119.7
C26—C25—C24	119.2 (2)	C27—C26—H26	119.7
C25—C26—C27	120.7 (2)	C26—C27—H27	119.9
C26—C27—C22	120.2 (2)	C22—C27—H27	119.9
O3'—S1'—O2'	121.04 (8)	C6'—N5'—H05'	116.1 (15)
O3'—S1'—N5'	106.17 (9)	S1'—N5'—H05'	115.4 (15)
O2'—S1'—N5'	105.68 (9)	N5'—C6'—H6'	108.2
O3'—S1'—C14'	107.42 (9)	C5'—C6'—H6'	108.2
O2'—S1'—C14'	108.00 (9)	C7'—C6'—H6'	108.2
N5'—S1'—C14'	107.94 (8)	C8'—C7'—H7'1	109.6
C2'—S2'—C21'	101.14 (9)	C6'—C7'—H7'1	109.6
C5'—O1'—C2'	102.12 (15)	C8'—C7'—H7'2	109.6
N3'—C2'—O1'	112.84 (15)	C6'—C7'—H7'2	109.6
N3'—C2'—S2'	126.02 (15)	H7'1—C7'—H7'2	108.1
O1'—C2'—S2'	120.99 (14)	C10'—C9'—H9'	119.4
C2'—N3'—N4'	105.81 (15)	C8'—C9'—H9'	119.4
C5'—N4'—N3'	106.39 (15)	C9'—C10'—H10'	120.0
N4'—C5'—O1'	112.82 (16)	C11'—C10'—H10'	120.0
N4'—C5'—C6'	128.53 (17)	C12'—C11'—H11'	120.4
O1'—C5'—C6'	118.62 (17)	C10'—C11'—H11'	120.4
C6'—N5'—S1'	121.64 (13)	C11'—C12'—H12'	119.6
N5'—C6'—C5'	114.01 (15)	C13'—C12'—H12'	119.6
N5'—C6'—C7'	108.00 (16)	C8'—C13'—H13'	119.9
C5'—C6'—C7'	109.93 (15)	C12'—C13'—H13'	119.9
C8'—C7'—C6'	110.48 (17)	C14'—C15'—H15'	120.3
C13'—C8'—C9'	118.82 (19)	C16'—C15'—H15'	120.3
C13'—C8'—C7'	120.67 (18)	C17'—C16'—H16'	119.4
C9'—C8'—C7'	120.44 (17)	C15'—C16'—H16'	119.4
C10'—C9'—C8'	121.10 (18)	C19'—C18'—H18'	119.3
C9'—C10'—C11'	119.9 (2)	C17'—C18'—H18'	119.3
C12'—C11'—C10'	119.2 (2)	C18'—C19'—H19'	120.4
C11'—C12'—C13'	120.7 (2)	C14'—C19'—H19'	120.4
C8'—C13'—C12'	120.2 (2)	C17'—C20'—H20D	109.5
C15'—C14'—C19'	120.34 (18)	C17'—C20'—H20E	109.5
C15'—C14'—S1'	119.89 (16)	H20D—C20'—H20E	109.5
C19'—C14'—S1'	119.75 (15)	C17'—C20'—H20F	109.5
C14'—C15'—C16'	119.49 (19)	H20D—C20'—H20F	109.5
C17'—C16'—C15'	121.26 (19)	H20E—C20'—H20F	109.5
C16'—C17'—C18'	118.27 (17)	C22'—C21'—H21C	108.6
C16'—C17'—C20'	121.69 (18)	S2'—C21'—H21C	108.6
C18'—C17'—C20'	119.99 (19)	C22'—C21'—H21D	108.6
C19'—C18'—C17'	121.31 (19)	S2'—C21'—H21D	108.6
C18'—C19'—C14'	119.22 (19)	H21C—C21'—H21D	107.6
C22'—C21'—S2'	114.75 (15)	C22'—C23'—H23'	119.8
C23'—C22'—C27'	118.94 (19)	C24'—C23'—H23'	119.8
C23'—C22'—C21'	118.76 (19)	C23'—C24'—H24'	119.8
C27'—C22'—C21'	122.25 (19)	C25'—C24'—H24'	119.8
C22'—C23'—C24'	120.4 (2)	C26'—C25'—H25'	120.4
C23'—C24'—C25'	120.4 (2)	C24'—C25'—H25'	120.4
C26'—C25'—C24'	119.2 (2)	C25'—C26'—H26'	119.7
C25'—C26'—C27'	120.5 (2)	C27'—C26'—H26'	119.7
C26'—C27'—C22'	120.5 (2)	C26'—C27'—H27'	119.7
C6—N5—H05	118.1 (17)	C22'—C27'—H27'	119.7
			
C5—O1—C2—N3	1.28 (19)	C5'—O1'—C2'—N3'	1.21 (19)
C5—O1—C2—S2	−175.38 (13)	C5'—O1'—C2'—S2'	−174.64 (13)
C21—S2—C2—N3	155.78 (17)	C21'—S2'—C2'—N3'	154.33 (17)
C21—S2—C2—O1	−27.96 (16)	C21'—S2'—C2'—O1'	−30.40 (16)
O1—C2—N3—N4	−1.7 (2)	O1'—C2'—N3'—N4'	−1.5 (2)
S2—C2—N3—N4	174.82 (13)	S2'—C2'—N3'—N4'	174.09 (13)
C2—N3—N4—C5	1.46 (19)	C2'—N3'—N4'—C5'	1.2 (2)
N3—N4—C5—O1	−0.72 (19)	N3'—N4'—C5'—O1'	−0.5 (2)
N3—N4—C5—C6	178.11 (16)	N3'—N4'—C5'—C6'	177.52 (17)
C2—O1—C5—N4	−0.26 (19)	C2'—O1'—C5'—N4'	−0.37 (19)
C2—O1—C5—C6	−179.19 (15)	C2'—O1'—C5'—C6'	−178.60 (15)
O2—S1—N5—C6	−41.23 (16)	O3'—S1'—N5'—C6'	−162.24 (14)
O3—S1—N5—C6	−170.22 (14)	O2'—S1'—N5'—C6'	−32.52 (16)
C14—S1—N5—C6	74.15 (16)	C14'—S1'—N5'—C6'	82.83 (16)
S1—N5—C6—C5	−69.23 (19)	S1'—N5'—C6'—C5'	−73.65 (19)
S1—N5—C6—C7	167.03 (13)	S1'—N5'—C6'—C7'	163.85 (13)
N4—C5—C6—N5	127.40 (19)	N4'—C5'—C6'—N5'	128.6 (2)
O1—C5—C6—N5	−53.8 (2)	O1'—C5'—C6'—N5'	−53.4 (2)
N4—C5—C6—C7	−110.7 (2)	N4'—C5'—C6'—C7'	−109.9 (2)
O1—C5—C6—C7	68.0 (2)	O1'—C5'—C6'—C7'	68.0 (2)
N5—C6—C7—C8	−174.88 (15)	N5'—C6'—C7'—C8'	178.48 (15)
C5—C6—C7—C8	59.7 (2)	C5'—C6'—C7'—C8'	53.5 (2)
C6—C7—C8—C13	−108.3 (2)	C6'—C7'—C8'—C13'	−107.2 (2)
C6—C7—C8—C9	70.2 (2)	C6'—C7'—C8'—C9'	69.8 (2)
C13—C8—C9—C10	−1.1 (3)	C13'—C8'—C9'—C10'	−0.2 (3)
C7—C8—C9—C10	−179.66 (18)	C7'—C8'—C9'—C10'	−177.21 (18)
C8—C9—C10—C11	0.8 (3)	C8'—C9'—C10'—C11'	0.3 (3)
C9—C10—C11—C12	−0.8 (3)	C9'—C10'—C11'—C12'	−0.1 (3)
C10—C11—C12—C13	1.0 (3)	C10'—C11'—C12'—C13'	−0.2 (3)
C11—C12—C13—C8	−1.3 (4)	C9'—C8'—C13'—C12'	−0.1 (3)
C9—C8—C13—C12	1.3 (3)	C7'—C8'—C13'—C12'	176.88 (19)
C7—C8—C13—C12	179.86 (19)	C11'—C12'—C13'—C8'	0.3 (3)
O2—S1—C14—C19	−4.05 (17)	O3'—S1'—C14'—C15'	−13.95 (17)
O3—S1—C14—C19	127.68 (15)	O2'—S1'—C14'—C15'	−146.02 (15)
N5—S1—C14—C19	−118.91 (15)	N5'—S1'—C14'—C15'	100.16 (16)
O2—S1—C14—C15	176.24 (14)	O3'—S1'—C14'—C19'	167.84 (14)
O3—S1—C14—C15	−52.03 (17)	O2'—S1'—C14'—C19'	35.77 (16)
N5—S1—C14—C15	61.38 (17)	N5'—S1'—C14'—C19'	−78.05 (16)
C19—C14—C15—C16	−0.2 (3)	C19'—C14'—C15'—C16'	1.3 (3)
S1—C14—C15—C16	179.55 (15)	S1'—C14'—C15'—C16'	−176.87 (14)
C14—C15—C16—C17	0.5 (3)	C14'—C15'—C16'—C17'	1.3 (3)
C15—C16—C17—C18	−1.1 (3)	C15'—C16'—C17'—C18'	−1.6 (3)
C15—C16—C17—C20	177.26 (18)	C15'—C16'—C17'—C20'	−178.99 (17)
C16—C17—C18—C19	1.5 (3)	C16'—C17'—C18'—C19'	−0.8 (3)
C20—C17—C18—C19	−176.93 (17)	C20'—C17'—C18'—C19'	176.64 (17)
C15—C14—C19—C18	0.5 (3)	C17'—C18'—C19'—C14'	3.4 (3)
S1—C14—C19—C18	−179.24 (14)	C15'—C14'—C19'—C18'	−3.6 (3)
C17—C18—C19—C14	−1.1 (3)	S1'—C14'—C19'—C18'	174.55 (14)
C2—S2—C21—C22	−62.79 (16)	C2'—S2'—C21'—C22'	−69.37 (15)
S2—C21—C22—C23	139.26 (17)	S2'—C21'—C22'—C23'	143.09 (16)
S2—C21—C22—C27	−42.1 (2)	S2'—C21'—C22'—C27'	−39.4 (2)
C27—C22—C23—C24	−0.7 (3)	C27'—C22'—C23'—C24'	−0.6 (3)
C21—C22—C23—C24	177.92 (19)	C21'—C22'—C23'—C24'	176.99 (18)
C22—C23—C24—C25	1.3 (3)	C22'—C23'—C24'—C25'	1.1 (3)
C23—C24—C25—C26	−0.8 (3)	C23'—C24'—C25'—C26'	−0.8 (3)
C24—C25—C26—C27	−0.1 (3)	C24'—C25'—C26'—C27'	0.1 (3)
C25—C26—C27—C22	0.6 (3)	C25'—C26'—C27'—C22'	0.4 (3)
C23—C22—C27—C26	−0.2 (3)	C23'—C22'—C27'—C26'	−0.2 (3)
C21—C22—C27—C26	−178.82 (18)	C21'—C22'—C27'—C26'	−177.65 (18)

### Hydrogen-bond geometry (Å, °)

**Table:**

*D*—H···*A*	*D*—H	H···*A*	*D*···*A*	*D*—H···*A*
N5—H05···N4^i^	0.79 (3)	2.22 (3)	3.003 (2)	178 (3)
N5'—H05'···N4'^i^	0.82 (3)	2.23 (3)	3.048 (2)	171 (2)
C21—H21B···O2'^ii^	0.99	2.32	3.249 (2)	155
C21'—H21D···O2^iii^	0.99	2.23	3.145 (2)	154
C19—H19···O3^iv^	0.95	2.50	3.352 (2)	150

Symmetry codes: (i) *x*, *y*−1, *z*; (ii) −*x*+1/2, *y*−1/2, −*z*+1; (iii) −*x*+1/2, *y*−1/2, −*z*; (iv) *x*, *y*+1, *z*.
